# A novel anisotropy template for an improved interpretation of elastic anisotropy data

**DOI:** 10.1038/s41598-023-43271-y

**Published:** 2023-09-27

**Authors:** Gama Firdaus, Manika Prasad, Jyoti Behura

**Affiliations:** Center for Rock & Fluid Multiphysics, Colorado School of Mines, Golden, CO 80401 USA

**Keywords:** Geophysics, Solid Earth sciences, Mineralogy

## Abstract

Tight unconventional rocks are characterized by the presence of laminations, preferentially oriented cracks, and an interconnected network of compliant minerals. Such anisotropic features can mechanically deform due to pressure depletion during production, leading to a human-induced change of elastic and fluid transport properties. Rock physics models allow us to better predict and assess stress- and direction-dependent elastic moduli of the rock, useful for horizontal stress estimates. However, elastic anisotropy can be challenging to measure and interpret. We have developed an anisotropy template that can be used to assess stress-dependent changes in elastic moduli and investigate rock textures. We present here the template construction using an effective medium model consisting of stiff and compliant layers and crack inclusions and evaluate the origin of stress-dependent stiffness changes in acoustic data from Berea, Bakken, Three Forks, and Mancos formations.

## Introduction

In tight unconventional formations, seismic anisotropy is due to intrinsic and extrinsic components, which can be found at multiple scales^1^. The intrinsic anisotropy can include sequences of thin layers^2–4^, lenticular clay sheets^5–7^ that are deposited subparallel to the bedding plane^8–11^, and aligned organic matter, pores, and cracks^4,12^. The extrinsic anisotropy of the rock can originate from aligned slit-like cracks and fractures^13–17^, which are due to drilling or coring operations^10^.

The spatial distribution, volumetric concentration, and stiffness of anisotropic components in rocks control the stress dependence of elastic properties^6,18,19^. During the production period, the dynamic interactions between pore pressure and overburden stress can cause compression of aligned compliant components and crack closure, leading to fluid flow path evolution. Rock fabric deformations have been characterized using anisotropic textural features in effective medium models for quantitative seismic interpretation^2,5,20–25^.

For example, the anisotropy parameters can be obtained from image logs, thin sections, computed tomography (CT) and scanning electron microscopy (SEM) images, and laboratory core measurements. However, such laboratory measurements are time- and resource-consuming and complicated. Field measurements of the complete elastic stiffness tensors of the rock are also rare^26^. Consequently, despite the need for multi-directional elastic stiffness required to model the seismic response, stiffness in only one direction is commonly available. Without constraints on the choice of parameters in rock physics models, the prediction of in situ seismic parameters is associated with significant uncertainties. We present a solution using an anisotropy template to assess rock anisotropy, texture, and deformation behavior based on mineralogy and acoustic data.

The anisotropy template is constructed by integrating several rock physics models, including Thomsen's anisotropy^3^, Backus averaging^2^, and Hudson's crack model^24^, which consider crack- and layer-induced anisotropy in the effective medium. These models were selected based on their simplicity, reliability, and common use. Note that the template can be used with any model relevant to the application.

The anisotropy template allows the user to understand the natural anisotropy of the medium and assess elastic moduli evolution along different directions due to changes in stress. Such insights into the texture or symmetry of the rock are essential to select the suitable matrix model and optimizing the hydraulic fracturing design. We show the application of the anisotropy template on laboratory-measured elastic data of the Berea, Bakken, Three Forks, and Mancos formations.

## Methodology

The effective elastic stiffness is controlled by the volumetric concentration and spatial distribution of stiff and compliant components in the rock. Stiff components are typically inorganic minerals, while clay minerals, organic matter, bedding-parallel cracks, and low aspect ratio pores comprise compliant components. Any alignment of the components leads to a directional dependence of physical properties, and changes in stress lead to non-uniform deformation. Thus, understanding the causal mechanisms for anisotropy and rock fabric deformation allows predictions of stress-dependent changes (e.g., during production). Anisotropic textures and rock deformation behavior can also dictate the initiation and propagation fractures and directional fluid transport. In the next sections, we discuss the construction and features of the anisotropy template and use it to assess elastic properties compliance of sedimentary rocks. Note that henceforth in this paper, we use the subscripts ∥ and ⊥ denote bedding-parallel and bedding-perpendicular directions, respectively.

### Textural anisotropy

**Figure 1:**
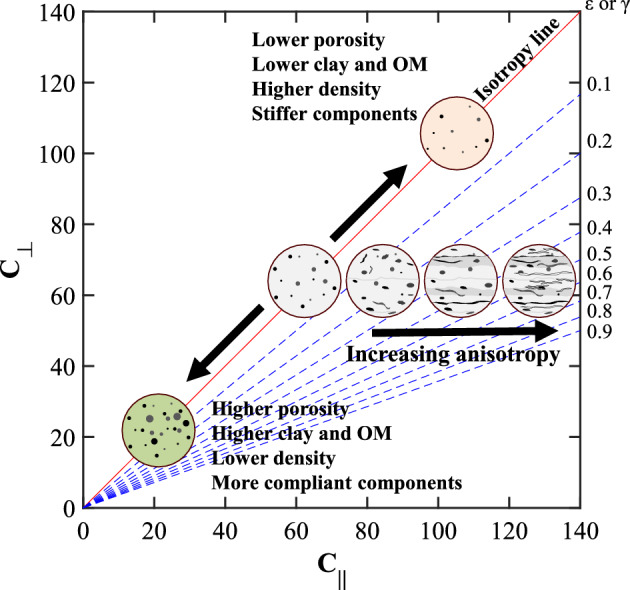
Schematic rock samples with various features and their respective location in the elastic stiffness cross plot. The red line marks the isotropy line, while the dashed lines mark increasing amounts of anisotropy.

Figure 1 shows a schematic of various rock fabrics and their effect on a cross plot of bedding-parallel (C_∥_) and bedding-perpendicular (C_⊥_) stiffnesses. The elastic stiffnesses of each direction are calculated in the form of C_XX_ = ρ x V^2^_XX_, where ρ is density, V is P- or S-wave velocity, and _XX_ represents ∥ and ⊥ directions. Also included in Fig. 1 are anisotropy lines (blue dashed lines) to reflect the effect of texture in the rock. We use Thomsen anisotropy parameters ε and γ to quantify anisotropy in transversely isotropic medium^3^.

For the isotropic case, variations in mineral composition and porosity move the stiffness along the diagonal, called the isotropy line. For the anisotropic case, the higher ∥ stiffness is larger than the ⊥ direction (C_∥_ > C_⊥_); therefore, the stiffness data points are distributed away from the isotropy line.

### Effective medium model

Two concepts used in the anisotropy template help understand textural controls or predict anisotropy with known texture and mineralogy: (a) the mineral components or the framework can be anisotropic, and (b) fractures or aligned pores can cause anisotropy. Since, as mentioned above, the anisotropy template can be used with any model, here we use the Backus averaging to model the anisotropic elastic properties of frame or layers^2^. We then introduce aligned cracks in this layered medium using Hudson's crack model^24^.

Both models assume that the textural features, layer thickness for Backus average and inclusion size and their separation distance for Hudson model, are smaller than the dominant seismic wavelengths. With ultrasonic frequency data, with wavelengths of about 3–6 mm, the anisotropy template is sensitive to the effective medium resulting from the arrangement and properties of core-scale layers, mineral alignments, and cracks. Using the anisotropy template with lower frequency data allows the user to assess anisotropic features at different measurement scales. We used the Backus average and the Hudson models based on their common usage, simplicity, and readily accessible inputs^27–30^. Alternative models can be used to construct the anisotropy, such as the displacement discontinuity method^5^, differential effective medium (DEM)^31^, self-consistent approximation (SCA)^21^, and crack models, including Eshelby^20^, Anderson et al.^22^, and Cheng^27^.

Note that there are several pitfalls related to the non-uniqueness in the models propagating into the anisotropy template. First, rock physics models may yield non-unique solutions because crack density, aperture dimension, and saturation can be combined in multiple ways to give the same answer. For example, the models might not distinguish between the presence of a single long fracture and the distribution of small fractures. Another ambiguity may come from the template’s inability to assess the moduli that are not parallel nor perpendicular to the bedding plane. However, we emphasize that such errors are common to all rock physics modeling applications and not specific to the template.

#### Backus averaging

**Figure 2:**
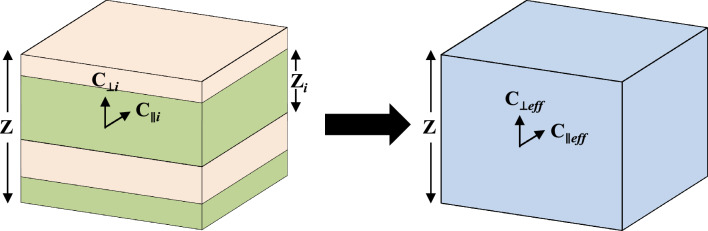
Elastic stiffness coefficients of individual layers are averaged to obtain an equivalent medium of thickness Z, where Z_i_ is the thickness of the *i*th horizontal layer. C_∥*i*_ and C_⊥*i*_ are the ∥ and ⊥ elastic stiffnesses of the *i*th layer in the layered medium, respectively. C_∥*eff*_ and C_⊥*eff*_ are the ∥ and ⊥ elastic stiffnesses of the effective medium calculated using Backus average.

We built frame properties for the anisotropy template with the Backus averaging technique^2^, where the effective medium of thickness, Z, consists of a stack of horizontal stiff and compliant layers with thickness, Z_i_ (Fig. 2). As a first approximation, individual layer properties, called the end member properties, are assumed isotropic and lie on the isotropy line in Fig. 1.

#### Hudson’s crack model

**Figure 3:**
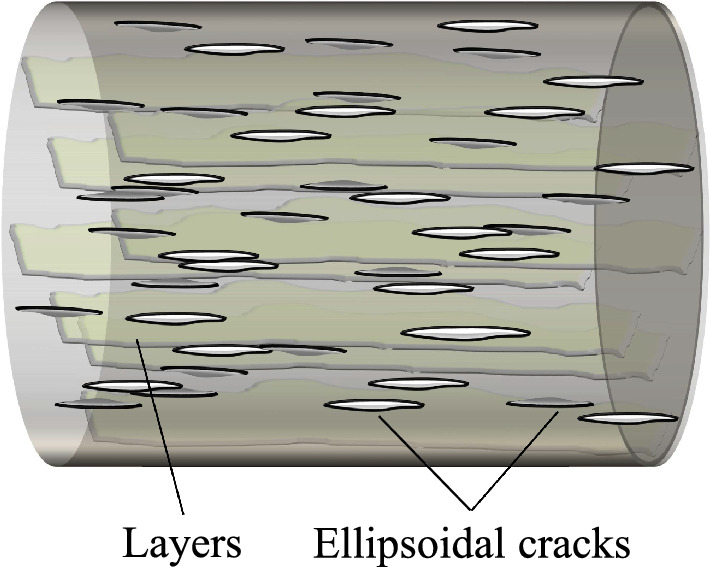
Crack-induced anisotropy due to the presence of ellipsoidal ∥ cracks.

To account for layer-parallel partings common in shales, we include air-filled penny-shaped ellipsoidal cracks in the layered medium (Fig. 3) and calculate the elastic stiffnesses using Hudson's crack model^24^. The effective elastic stiffness is calculated as C_ij_^eff^ = C_ij_^0^ + C_ij_^1^ + C_ij_^2^, where C_ij_^0^ is the effective elastic stiffness of the isotropic background rock, and C_ij_^1^ and C_ij_^2^ are the first and second-order corrections, respectively.

The Hudson crack model has a limited crack density range, less than 0.19, for very small aspect ratio values. For crack density values greater than the limitation, the modeled elastic stiffnesses increase, which is not physical^27^. For the application presented here, a crack density below 0.16 adequately explained the data.

### Anisotropy template construction workflow

**Figure 4:**
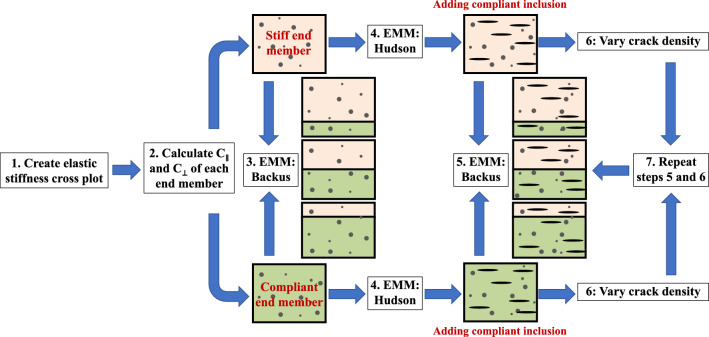
Workflow of the construction of the anisotropy template. C_∥_, C_⊥_, and EMM are the ∥ stiffness, ⊥ stiffness, and effective medium model, respectively.

To facilitate the use of the template, we present the workflow (Fig. 4) along with numerical examples for each step. The data needed to create the template are mineralogy, which can be estimated from multi-mineral log analysis or measured in the lab, density, and velocity of each mineral.

1. Create a cross plot with C_∥_ on the x-axis and C_⊥_ on the y-axis.
2. Add isotropy and anisotropy lines between 0 and 0.9 (Fig. 5a).
3. Calculate C_∥_ and C_⊥_ of the stiff and the compliant end members based on formation mineralogy. In the example shown in Fig. 5a, we used the Voigt-Reuss-Hill (VRH) average^32^ of the elastic stiffnesses and marked them as "stiff" and "compliant". Henceforth, in the context of the background medium, we use the term end member to denote both the stiff and the compliant components of the formation.
  The choice of end members is not restricted to single minerals. End member moduli may be calculated as averages of mineral moduli with similar stiffnesses. Table S1 in the *Supporting Information* shows possible compositions and properties of the stiff and compliant end members used to build Fig. 5a. For the organic-rich shales considered here, the compliant end member is an organo-clay composite where the clay and organic matter are mixed isotropically^33,34^, whereas the stiff end member is a mix of quartz, calcite, and dolomite. Both end members are assumed isotropic, and the stiffnesses are calculated using VRH. Alternatively, instead of partitioning minerals into stiff and compliant components, the Reuss average may represent the compliant end member and the Voigt average the stiff end member. Similarly, the Hashin-Strikman upper and lower bounds model also allows calculating end member moduli. The properties of each mineral include non-crack porosity and are shown in Table S2 in the *Supporting Information*.

**Figure 5:**
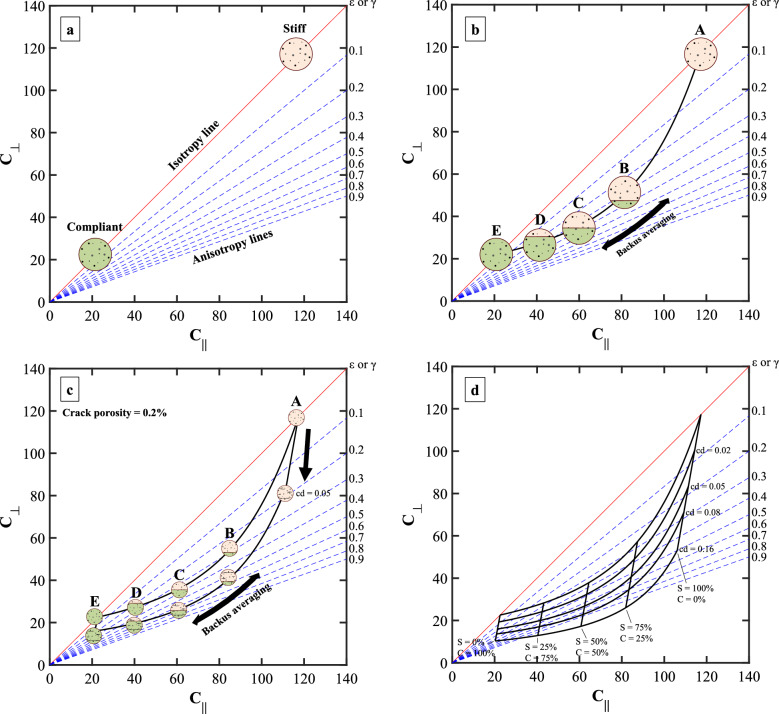
(**a**) Elastic stiffness cross plot that shows isotropy (red) and anisotropy lines (blue). Stiff and compliant end members lie on the isotropy line. (**b**) Elastic stiffnesses of the effective medium calculated using Backus averaging. The model line represents the cumulative concentrations of stiff and compliant layers shown in Table S3. (**c**) Addition of cracks to the layered medium modeled using Hudson's crack model. Here, the layered medium has crack density = 0.05 and crack porosity = 0.2%. (**d**) Anisotropy template with different compositions and crack densities (cd). Volume concentrations of the stiff layer (S) and the compliant layer (C) are shown below iso-volume lines.
  Note that anisotropic clay minerals can also be incorporated into the end member. In this case, the preferred orientation of clay minerals can increase the magnitude of the anisotropy^35^. Consequently, the compliant end member and the model lines in the template would deviate from the isotropy line.
4. Create a layered solid composed of parallel stiff and compliant layers using an effective medium model. For example, in Fig. 5b, we used the Backus average for varying proportions of stiff and compliant layers, as represented in Table S3.
5. Add ∥ cracks to the effective medium solid using a crack model. For example, in Fig. 5c, we used Hudson's crack model up to the second order to introduce air-filled ∥ penny-shaped cracks with crack density = 0.05 and crack porosity = 0.2%.
  The stepwise addition of cracks exhibited here has also been performed by other authors^25,36^. However, Nishizawa and Yoshino^36^ only focused on models that can be applied to crustal rocks, where crystal shape and orientation are dominant factors for the anisotropy.
6. Use Backus average again to mix end members that contain inclusions (cracks).
7. Repeat Step 5 by varying the crack density. For example, Fig. 5c shows end member values for crack density 0.05.
8. Create a layered solid using Backus average with the cracked end members mixed in different proportions. In Fig. 5d, the curved model lines represent crack densities = 0.02, 0.05, 0.08, and 0.16. The near-vertical iso-volume lines are where the volumetric concentration of the stiff and compliant layers is the same between multiple modeled curves.

### Stress sensitivity

**Figure 6:**
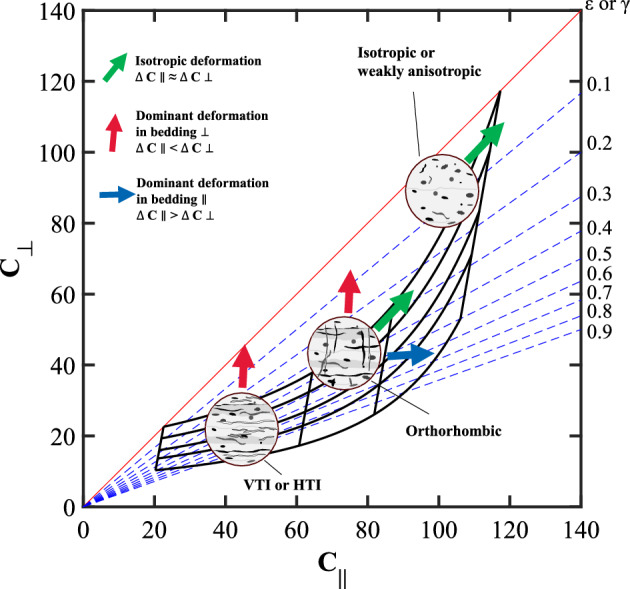
Typical deformations that occur in the anisotropy template due to stress change, causing various effective medium with different textures to follow the trend of to the red, green, or blue arrow. The arrows represent deformation slopes created for relative changes in ΔC_∥_ and ΔC_⊥_, where ΔC_∥_ and ΔC_⊥_ denote the stress-dependent increment of elastic stiffness coefficient in the ∥ and ⊥ direction, respectively.

Changes in stress increase grain-to-grain contacts and compress compliant components^6^. In addition to the compression of layered kerogen and clay, crack closure is a dominant force that leads to directional deformation of the rock. In the case of oriented stress or aligned textures, the deformations in ∥ and ⊥ directions are not equal: the ⊥ direction experiences higher compaction than the ∥ direction. This section discusses using the anisotropy template to assess the causes of stress- and direction-dependent stiffness changes. Figure 6 presents schematic illustrations of the effect of stress on cracks oriented along and across bedding planes.

#### Isotropic or weakly anisotropic medium

The increase in stiffness with stress in an isotropic or weakly anisotropic medium depends on the volume and the orientation of the compliant component. In porous media, a lack of anisotropy implies the presence of sub-rounded micropores that experience isotropic deformation with increasing hydrostatic load. Thus, deformations in ∥ and ⊥ directions will be approximately equal and ΔC_∥_ ≈ ΔC_⊥_, where ΔC_∥_ and ΔC_⊥_ denote the change in elastic stiffness with stress in the ∥ and ⊥ direction, respectively. In rocks composed of stiff components with low porosity, stress sensitivity will be low, and ΔC_∥_ and ΔC_⊥_ will be negligible.

#### Vertical transverse isotropy (VTI) and horizontal transverse isotropy (HTI) medium

For rocks with VTI and HTI symmetry, the compression of ∥ compliant components significantly increases C_⊥_ leading to ΔC_∥_ < ΔC_⊥_ with increasing stress. Consequently, the crack density and the anisotropy decrease in such "high strain regimes"^37^.

#### Orthorhombic medium

The deformation behavior of rocks depends heavily on their texture. In the case of orthorhombic symmetry, changes in stiffness with stress depend on the most compliant components. If the compression of ∥ components, such as clay and kerogen layers, is dominant, increasing the confining stress leads to ΔC_∥_ < ΔC_⊥_. If the deformation of compliant components, such as fractures, aligned orthogonal to the bedding plane is more significant, the dominant deformation will be in the ∥ direction, producing ΔC_∥_ > ΔC_⊥_. Of course, in nature, rocks often experience a combination of directional deformations and moduli changes and might yield a cumulative ΔC_∥_ ≈ ΔC_⊥_. The anisotropic template can be used to investigate possible textures with known stress-dependent moduli changes. Similarly, with known textures, the anisotropy template can be used to assess and constrain the range of modulus changes due to stress.

## Data analysis

In the context of this paper, it is important to acknowledge certain limitations associated with the scope and capabilities of the anisotropy template. The analysis presented here primarily focuses on the characterization of ∥ and ⊥ moduli as the key parameters for assessing rock anisotropy. Consequently, we have not included an exploration of C_13_, delta, or off-axis measurements in the current study. These parameters, although valuable in understanding anisotropy from various angles, are beyond the immediate scope of this research endeavor. It is crucial to recognize that attempting to incorporate off-axis data, acquired from inclined formation beds or deviated well trajectories, within our template might yield inaccurate anisotropy calculations and potentially result in an underestimation of crack density within the formation.

By focusing on ∥ and ⊥ moduli, the anisotropy template enables users to attain a comprehensive understanding of the range and evolution of anisotropy under stress variations. As such, any supplementary off-axis measurements should ideally align with the anisotropy range derived from these fundamental moduli. Moreover, it is worth noting that the applicability of the anisotropy template is primarily tailored to the analysis of anisotropy in relatively straightforward lithologies, such as VTI rocks. For lithologies featuring complex geometries, like fractured carbonates, the template may not yield accurate results.

To enhance the versatility and expand potential applications of the anisotropy template, several paths for future work are recommended. First and foremost, the inclusion of C_13_ and delta measurements is highly advisable, as these parameters play a pivotal role in comprehensive anisotropy assessment, imaging analysis, and geomechanical applications. Furthermore, exploring the effects of fluid saturation is an important direction for extension. Constructing an anisotropy template based on alternative effective medium models, such as the displacement discontinuity method and fluid-filled models, can be beneficial. Lastly, to broaden the scope and relevance of this research for practical geophysical and geomechanical studies, exploring moduli obtained from well logs as a function of depth is a promising path for future research.

In the following sections, we present the usage of the anisotropy template. Here we use laboratory-measured elastic moduli to assess textures and stress-dependent changes at the core scale. Subsurface phenomena explained with the template will depend on the scale of the data used. For example, overburden compression and pore pressure depletion acting upon a producing formation can lead to pore structure deformation of the rock. Similarly, sensitivity to crack closure, mineral compression, or compliant textural alignments can be detected from elastic stiffnesses in seismic data or earthquake seismology.

**Figure 7:**
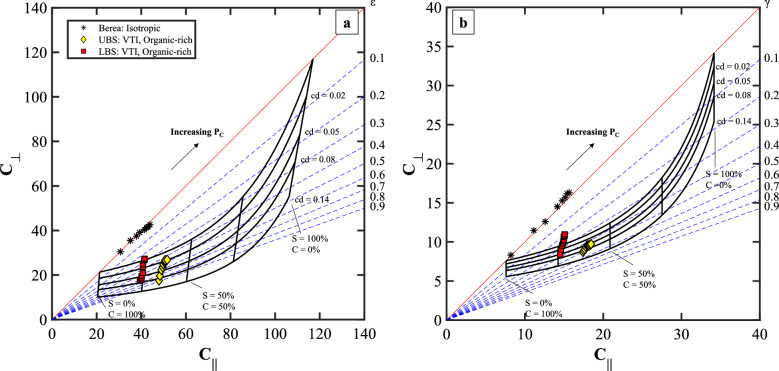
The developed anisotropy template for (**a**) compressional vti and (**b**) shear wave stiffness data for Bakken, Upper Bakken Shale (UBS), and Lower Bakken Shale (LBS). Acoustic data are available in Firdaus and Prasad^38^. Each point represents the calculated elastic stiffness coefficient at a pressure step of the respective sample. S, C, cd, and P_C_ are the volumetric concentration of the stiff layer, the volumetric concentration of the compliant layer, crack density, and confining pressure, respectively.

**Figure 8:**
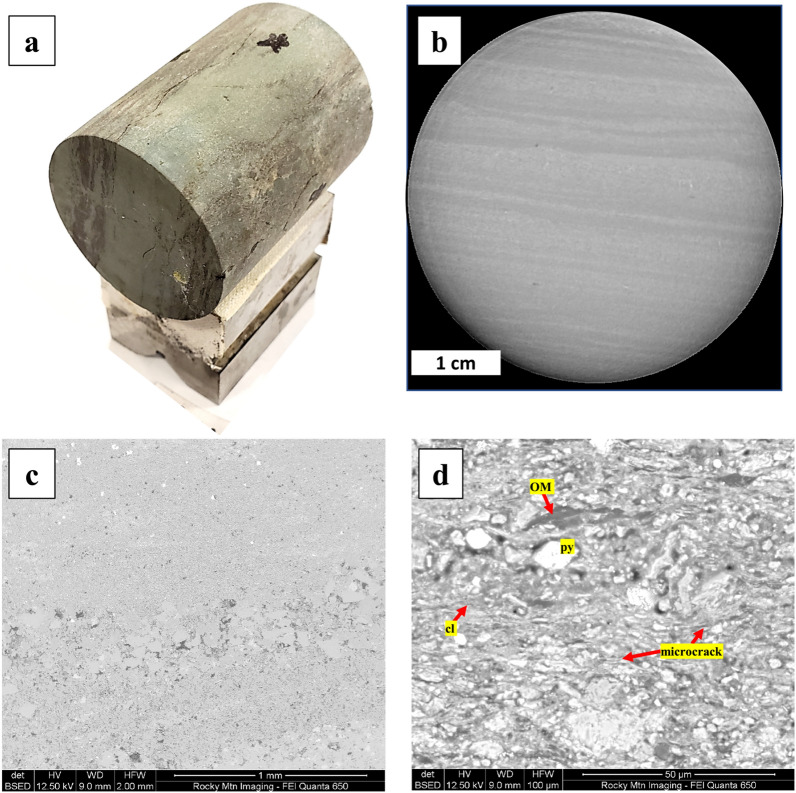
(**a**) Three Forks 1.5-in. core, (**b**) micro-CT image of Mancos, back-scattered SEM images of (**c**) Three Forks, and (**d**) Lower Bakken Shale sample.

**Figure 9:**
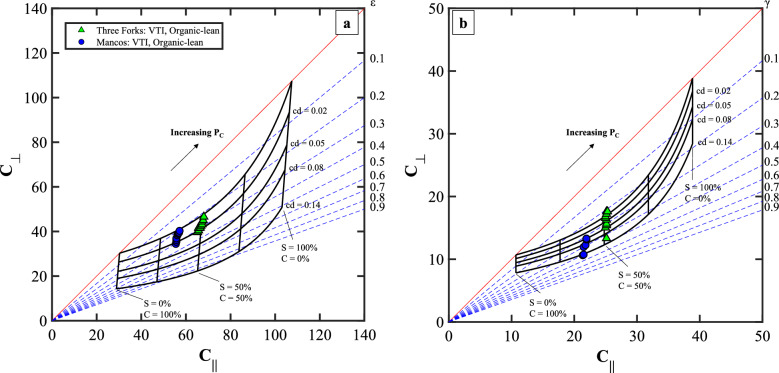
The developed anisotropy template for (**a**) compressional wave and (**b**) shear wave of Three Forks and Mancos formations. S, C, cd, and P_C_ are the volumetric concentration of the stiff layer, the volumetric concentration of the compliant layer, crack density, and confining pressure, respectively.

Figures 7 and 8 present the elastic stiffness coefficients of cores from Berea, Upper Bakken Shale (UBS), Lower Bakken Shale (LBS), Three Forks, and Mancos formations. The multidirectional ultrasonic P- and S- wave velocities were measured at elevated hydrostatic confining stress up to 27.6 MPa^10^. Elastic properties and mineralogy data are available in Firdaus and Prasad^38^. In Fig. 9, we show evidence of layering and heterogeneity of the rocks analyzed in the anisotropy template. Although, as expected, elastic stiffnesses increase and anisotropy decrease with the increase of hydrostatic load, representative samples from each formation exhibit unique behavior in the anisotropy template based on their textural differences. The lab data provides an example of how to apply the template if there is similar information available for geophysical borehole logs.

### Case: Isotropic

The Berea formation is categorized as isotropic with well-sorted sub-rounded quartz grains and a homogeneous microstructure^39,40^. The compressional and shear stiffnesses, C_∥_ and ΔC_⊥_, lie on the isotropy line, and the moduli experience a similar rate of change with pressure (ΔC_∥_ ≈ ΔC_⊥_) (Fig. 7a and b). The load increase leads to an increase in the contact interface between grains and homogeneous deformation of microcracks.

### Case: VTI, organic-rich

In Fig. 7a and b, the Upper Bakken Shale (UBS) and Lower Bakken Shale (LBS) formations are denoted by yellow and magenta diamonds. The stiffnesses of the UBS and LBS are low due to the high presence of compliant components, such as kerogen, which can be as high as 21 vo l%^10^. As a result, the shale samples exhibit high sensitivity to pressure, especially in the ⊥ direction (ΔC_∥_ < ΔC_⊥_). Based on this deformation behavior, the Bakken shales can be categorized as VTI rocks.

At low tested confining pressure (3.4 MPa), ε and γ are as high as 90% and 70%, respectively. Since the rock samples are unpreserved, coring-induced cracks may have developed and increased the anisotropy^15^. At the highest tested confining pressure (27.6 MPa), ε and γ values remain above 20% due to intrinsic anisotropy. This high sensitivity to pressure behavior has also been reported in other Bakken shale measurements^4,7,10,11,18^, which is primarily due to the closure low aspect ratio microcracks that reside in the clay bodies (Fig. 8), as well as the compression of compliant components (e.g., kerogen and illite–smectite laminations) that are oriented parallel to the bedding plane^6,8,13,15^.

### Case: VTI, clay-rich and organic-lean

Three Forks and Mancos formations depicted in Fig. 9a and b have a moderate concentration of clay (> 20%) and very low organic content (< 2%). The main observable features are C_∥_ > C_⊥_ and significant pressure-dependent stiffness changes in the ⊥ direction (ΔC_∥_ < ΔC_⊥_) for both compressional and shear waves. Based on the data distribution in the template, we conclude that both Three Forks and Mancos have VTI symmetry. Additionally, the increase of confining pressure caused the ε value to decrease by 10%, whereas the γ value had a more significant change, approximately up to 30% decrease. Figure 8 shows evidence of horizontal layers of clay that alternate with the dominating calcite/dolomite matrix at multiple scales. The contribution of clay compression to the change of elastic moduli, however, is relatively small compared to the role of crack closure that are aligned to the bedding plane.

We notice, however, that the P and S elastic moduli for a given sample do not fall on the same position on the template crack density grid in Figs. 7 and 9. The misfit may result from the assumption of a crack orientation parallel to the bedding plane, while cracks orthogonal to the bedding plane might exist in the rock. Additionally, the ambiguity may be due to the incomplete fluid removal in the core–fluid effects are not modeled in the template.

## Conclusions

We have developed an anisotropy template that can be utilized to assess elastic moduli along different directions and evaluate the pressure dependency of the rock fabric. We show how the anisotropy template can be utilized to capture the texture or symmetry of the rock with known stress-dependent moduli changes. The anisotropy template used with geophysical data from Bakken shales, Three Forks, and Mancos formations reveals that the increase of hydrostatic stress leads to a significant increase of elastic moduli in the ⊥ direction, which indicates that such rocks have VTI symmetry. The template application can benefit geoscientists and engineers in understanding the relevance of cracks and improving the characterization of anisotropic rocks. Capturing the right texture or symmetry of the rock can lead to a more accurate matrix model and ultimately optimize hydraulic fracturing design.

### Supplementary Information

Supplementary Tables.

## Data Availability

Acoustic data used in this study are available from the following reference: 10.1594/PANGAEA.933121. (i) Composition and average properties of the stiff and compliant end members, and (ii) density and velocity of minerals used in this paper are included in the Supporting Information.
