# Supplementary material for: A novel anisotropy template for an improved interpretation of elastic anisotropy data
Source: Sci Rep. 2023 Sep 27;13:16160. doi: 10.1038/s41598-023-43271-y (PMC10533563; doi:10.1038/s41598-023-43271-y)
Supplement: Supplementary file 1 — Supplementary Tables. [file 41598_2023_43271_MOESM1_ESM.docx]

SUPPORTING INFORMATION

A Novel Anisotropy Template for an Improved Interpretation of Elastic Anisotropy Data

Gama Firdaus^1^*, gamafirdaus@mines.edu

Manika Prasad^1^*, mprasad@mines.edu

Jyoti Behura^1^, jbehura@mines.edu

^1^Center for Rock & Fluid Multiphysics, Colorado School of Mines, Golden, Colorado 80401, United States

**CORRESPONDING AUTHOR**

*Gama Firdaus: gamafirdaus@mines.edu

*Manika Prasad: mprasad@mines.edu

Table S1 shows possible compositions and properties of the stiff and compliant end members used to build Fig. 5a. The volumetric concentration of minerals that constitute the stiff and compliant end members for (i) case: VTI, organic-rich rocks and (ii) case: VTI, clay-rich and organic-lean rocks are represented in Table S2. Table S3 shows the mineral properties to construct the end members.

| **Table S1. Volumetric concentration and moduli (compressional) of stiff and compliant layers in mixtures A-E.** | | | | |
| --- | --- | --- | --- | --- |
| Points (Figure 5b) | Stiff component volume | Compliant component volume | C_∥_ (GPa) | C_⊥_ (GPa) |
| **A** | 100% | 0% | 117.3 | 117.3 |
| **B** | 75% | 25% | 87.4 | 57.2 |
| **C** | 50% | 50% | 64.4 | 37.9 |
| **D** | 25% | 75% | 43.2 | 28.3 |
| **E** | 0% | 100% | 22.6 | 22.6 |

| **Table S2. Composition and average properties of the stiff and compliant end members.** | | | | | | |
| --- | --- | --- | --- | --- | --- | --- |
| Case | End member | Mineral | Fraction | Average Density | V_P_ (VRH) | V_S_ (VRH) |
|  |  |  |  | (g/cc) | (km/s) | (km/s) |
| VTI, organic-rich | Stiff | Quartz | 15% | 2.71 | 6.57 | 3.55 |
|  |  | Calcite | 80% |  |  |  |
|  |  | Dolomite | 5% |  |  |  |
|  | Compliant | Clay | 60% | 2.15 | 3.15 | 1.88 |
|  |  | Kerogen | 40% |  |  |  |
| VTI, clay-rich and organic-lean | Stiff | Quartz | 55% | 2.69 | 6.32 | 3.8 |
|  |  | Calcite | 40% |  |  |  |
|  |  | Dolomite | 5% |  |  |  |
|  | Compliant | Clay | 90% | 2.47 | 3.5 | 2.08 |
|  |  | Kerogen | 10% |  |  |  |

| **Table S3. Density and velocity of minerals used in this paper to construct the anisotropy template.** | | | | |
| --- | --- | --- | --- | --- |
| Mineral | Density | V_P_ | V_S_ | References |
|  | (g/cc) | (km/s) | (km/s) |  |
| Quartz | 2.65 | 6.05 | 4.09 | Carmichael (1989) |
| Calcite | 2.71 | 6.64 | 3.44 | Simmons (1965) |
| Dolomite | 2.87 | 7.34 | 3.96 | Mavko et al. (2020) |
| Clay | 2.55 | 3.81 | 1.88 | Han et al. (1986) |
| Kerogen | 1.5 | 2.54 | 1.53 | Yan and Han (2013) |
